# Genomics and Comparative Genomic Analyses Provide Insight into the Taxonomy and Pathogenic Potential of Novel *Emmonsia* Pathogens

**DOI:** 10.3389/fcimb.2017.00105

**Published:** 2017-03-31

**Authors:** Ying Yang, Qiang Ye, Kang Li, Zongwei Li, Xiaochen Bo, Zhen Li, Yingchun Xu, Shengqi Wang, Peng Wang, Huipeng Chen, Junzhi Wang

**Affiliations:** ^1^Academy of Military Medical SciencesBeijing, China; ^2^Department of Biotechnology, Beijing Institute of Radiation MedicineBeijing, China; ^3^Department of Biological Product Control, National Institutes for Food and Drug ControlBeijing, China; ^4^Key Laboratory of the Ministry of Health for Research on Quality and Standardization of Biotech ProductsBeijing, China; ^5^Center for Hospital Infection Control, Chinese PLA Institute for Disease Control and PreventionBeijing, China; ^6^Division of Medical Microbiology, Peking Union Medical College HospitalBeijing, China

**Keywords:** *Emmonsia* species, novel fungal pathogens, comparative genomic analyses, taxonomy, pathogenicity

## Abstract

Over the last 50 years, newly described species of *Emmonsia*-like fungi have been implicated globally as sources of systemic human mycosis (emmonsiosis). Their ability to convert into yeast-like cells capable of replication and extra-pulmonary dissemination during the course of infection differentiates them from classical *Emmonsia* species. Immunocompromised patients are at highest risk of emmonsiosis and exhibit high mortality rates. In order to investigate the molecular basis for pathogenicity of the newly described *Emmonsia* species, genomic sequencing and comparative genomic analyses of *Emmonsia* sp. 5z489, which was isolated from a non-deliberately immunosuppressed diabetic patient in China and represents a novel seventh isolate of *Emmonsia*-like fungi, was performed. The genome size of 5z489 was 35.5 Mbp in length, which is ~5 Mbp larger than other *Emmonsia* strains. Further, 9,188 protein genes were predicted in the 5z489 genome and 16% of the assembly was identified as repetitive elements, which is the largest abundance in *Emmonsia* species. Phylogenetic analyses based on whole genome data classified 5z489 and CAC-2015a, another novel isolate, as members of the genus *Emmonsia*. Our analyses showed that divergences among *Emmonsia* occurred much earlier than other genera within the family Ajellomycetaceae, suggesting relatively distant evolutionary relationships among the genus. Through comparisons of *Emmonsia* species, we discovered significant pathogenicity characteristics within the genus as well as putative virulence factors that may play a role in the infection and pathogenicity of the novel *Emmonsia* strains. Moreover, our analyses revealed a novel distribution mode of DNA methylation patterns across the genome of 5z489, with >50% of methylated bases located in intergenic regions. These methylation patterns differ considerably from other reported fungi, where most methylation occurs in repetitive loci. It is unclear if this difference is related to physiological adaptations of new *Emmonsia*, but this question warrants further investigation. Overall, our analyses provide a framework from which to further study the evolutionary dynamics of *Emmonsia* strains and identity the underlying molecular mechanisms that determine the infectious and pathogenic potency of these fungal pathogens, and also provide insight into potential targets for therapeutic intervention of emmonsiosis and further research.

## Introduction

Historically, the clinical relevance of the genus *Emmonsia* was limited to a very rare and unusual pulmonary disease, adiaspiromycosis, which primarily affects rodents, fossorial mammals, and their predators, but is rarely documented in humans (Sigler, 2005). Two classical species, *Emmonsia crescens* (*E. crescens*) and *Emmonsia parva* (*E. parva*), have been identified as the etiological agents of this disease (Anstead et al., 2012). However, newly discovered species of *Emmonsia*-like fungi, with phylogenetic and clinical affinities to *Blastomyces* and *Histoplasma*, have been implicated as causes of systemic human mycoses globally over the last 50 years. Their ability to produce a thermally-dependent yeast-like phase rather than adiaspores, and cause disseminated infections differentiates them from classical *Emmonsia* species. Immunocompromised patients are predominantly susceptible to infection, and case-fatality rates have approached 50% (Schwartz et al., 2015a). Seven novel *Emmonsia*-like fungi have been reported worldwide thus far. The isolates all share morphological and pathological characteristics including the production of yeast-like cells rather than adiaspores at high temperature, causing extra-pulmonary dissemination following infection and causing profound impairment of cell-mediated immunity (Schwartz et al., 2015b). However, each isolate exhibits unique clinical characteristics and can be identified as “novel” based on sequence analyses of internal transcribed spacer (ITS) genomic regions (Schwartz et al., 2015b). However, whether these novel species have only recently emerged as human pathogens, or alternatively, that previous infections were simply underestimated, remains debatable. Remarkable increases in the number of disseminated emmonsiosis cases reported in South Africa coincide with the introduction of molecular identification tools, providing support for the notion that previous infections went unnoticed due to the lack of adequate detection methodologies (Kenyon et al., 2013; Schwartz et al., 2015b).

The taxonomic classification of *Emmonsia* has been subject to debate even prior to the discovery of the novel species. *E. parva* was found to be more closely related to the *Blastomyces* genus than to *E. crescens* in two phylogenetic studies based on 18S ribosomal DNA sequences (Peterson and Sigler, 1998; Untereiner et al., 2014). With regards to the phylogenetic position of *E. crescens*, one analysis placed it as sister to the *Paracoccidioides* (Peterson and Sigler, 1998), while in another analysis it was placed in a clade with *Blastomyces* and *E. parva* (Untereiner et al., 2014). Furthermore, other preliminary phylogenetic analyses based on several housekeeping genes suggested that the majority of the new human-associated *Emmonsia*-like fungi may group together in a derived monophyletic clade in the Ajellomycetaceae family (Schwartz et al., 2015b). Robust genome sequencing and analyses of *Emmonsia*-like species could resolve their phylogenetic relationships and provide a foundation for understanding the molecular basis for infectious potency of *Emmonsia* strains.

Recently, the genome sequences of two classical *Emmonsia* species, *E. crescens* and *E. parva*, have become publicly available (Muñoz et al., 2015). These strains were isolated from a weasel in Ravelli County, Montana and from the lungs of a rodent (*Arvicola terrestris*) in Norway, respectively. The genome sequence of a third *Emmonsia* (*Emmonsia* sp. CAC-2015a; also known as *E. africana* CBS 136260) is also publically available (Genbank accession number LGUA00000000). *Emmonsia* sp. CAC-2015a was isolated from a HIV patient skin biopsy from South Africa and represents the fifth novel *Emmonsia*-like fungus isolate. *Emmonsia* sp. 5z489 (CBS 124587 = CGMCC2.4011), the seventh novel *Emmonsia*-like fungus, was isolated in 2005 from a diabetic patient from Shanxi, China, who had disseminated emmonsiosis (Wang et al., 2009, 2017). In contrast to most documented emmonsiosis in immunosuppressed patients, *Emmonsia* sp. 5z489 was isolated from a patient with non-deliberate immunosuppression, apart from the presence of diabetes mellitus.

Here, the genome of *Emmonsia* sp. 5z489 was sequenced using a combination of the Pacific Biosciences RS II and Illumina MiSeq platforms, which allowed for a higher level of genome quality and a greater level of genome completeness than is present in existing *Emmonsia* genomes. Additionally, the global state of DNA methylation patterns was investigated, the phylogenetic position of *Emmonsia* sp. 5z489 was assessed and unique genomic features were analyzed via comparative genomic analyses. Moreover, potential virulence factors in species 5z489 and CAC-5015a that may be important for invasion and diffusion are reported and characterized through comparative genomic analyses. Our study provides a foundation for the identification of the underlying molecular mechanisms that differentiate the infectious potentials of newly described *Emmonsia* strains.

## Materials and methods

### Culture conditions and DNA isolation

*Emmonsia* sp. 5z489 was grown on Brain Heart Infusion Agar media at 37°C for 15 days. Genomic DNA for whole-genome sequencing was extracted from the culture using the DNeasy Plant Mini kit (QIAGEN Co., Ltd, Hamburg, Germany) according to the manufacturer's instructions.

### Genomic analyses

A combined sequence platform strategy was used for *Emmonsia* sp. 5z489 genome sequencing. Illumina MiSeq (Illumina Inc., San Diego, CA, USA) and Pacific Biosciences RS II (Pacific Biosciences, Menlo Park, CA, USA) sequencing platforms were used in combination, whereby the short-read sequence data from the Illumina platform were used to increase the resolution and quality of the longer-read PacBio assembly sequences. The Nextera® XT DNA Sample Preparation Kit (Illumina Inc., San Diego, CA, USA) was used to prepare the Illumina library according to the manufacturer's instructions. One 250 bp paired-end (PE) sequencing library was constructed with an insert size of ~400 bp. For Pacific Biosciences RS II sequencing, a 20-kbp Single Molecule Real Time (SMRT) bell library was prepared from sheared genomic DNA using a 20-kbp template library preparation workflow. The PacBio RS II sequencing platform was used to conduct SMRT sequencing using C3 sequencing chemistry and the P5 polymerase with 16 SMRT cells.

The hybrid genome was assembled using the Hierarchical Genome Assembly Process (HGAP). *De novo* assembly of the PacBio read sequences was performed using continuous long reads (CLR), followed by the HGAP workflow (PacBioDevNet; Pacific Biosciences) as available in the SMRT Analysis V. 2.1 software package. The HGAP workflow comprises preassembly, *de novo* assembly with the Celera Assembler (CA), and assembly polishing using Quiver. For the preassembly step, CA software V. 7.0 was used. Error correction for the raw data generated by the PacBio RS II platform was performed using the PacBioRs_PreAssembler with 1 module, a default minimum subread length of 500 bp, a minimum read quality of 0.80, and a minimum subread length of 7,500 bp. The MiSeq read sequences were mapped using the Burrows-Wheeler Aligner (BWA; V. 0.7.5a) in order to improve the accuracy of the assembled sequence from HGAP (Li and Durbin, 2009, 2010). SNPs and indels were called and corrected using SAMtools V. 0.1.18 (Li et al., 2009) and an in-house script. The genome of *Emmonsia* sp. 5z489 was deposited in Genbank under the accession number MOWL00000000.

### Gene prediction and annotation

Protein-coding genes in the *Emmonsia* sp. 5z489 genome were predicted using AUGUSTUS (Stanke et al., 2006). Functional annotations of predicted genes were assessed according to their similarity to genes and proteins in the NCBI non-redundant (nr) database and using *E*-value cut-offs of 1e-5 and ≥40% amino acid identity to assign gene identities. All genes were classified based on gene ontology (GO) terms using BLAST2GO (Conesa et al., 2005). For pathway analysis, genes were annotated with the Kyoto Encyclopedia of Genes and Genomes (KEGG) database using blastKOALA (Ogata et al., 1999), and assigned KO identifiers which were used to map KEGG pathways using KEGG mapper. Putative gene functions were classified according to the Eukaryotic Orthologous Groups (KOG) database. Pfam analysis was performed using a batch sequence search against the Pfam database (Finn et al., 2014) with an *E*-value cutoff of ≤ 1e-5.

### Orthologous gene families and phylogenetic analyses

The Markov clustering program OrthoMCL (Li et al., 2003) was used to identify orthologous genes among the four *Emmonsia* species. Peptide sequences were subjected to an all-vs.-all BLASTp search against the nr database with an *E*-value threshold of ≤ 1e-5. Putative orthologs were then clustered with OrthoMCL using an inflation value of 1.5. A maximum-likelihood phylogenetic tree was constructed based on the sequences of 192 single-copy orthologs using RAxML (Stamatakis, 2006).

### Prediction of pathogenicity-related proteins and the secretome

To predict putative pathogenicity-related proteins, BLASTp searches were conducted against the Pathogen-Host Interaction (PHI) database (Winnenburg et al., 2006) with an *E*-value threshold of ≤ 1e-5. Additional BLASTp searches were conducted against the Database of Fungal Virulence Factors (DFVF; Lu et al., 2012) with the *E*-value threshold of ≤ 1e-10. Secretory proteins were identified as described by Verma et al. (2016).

## Results

### Genome features and DNA methylation patterns of *Emmonsia* sp. 5z489

**Figure 1:**
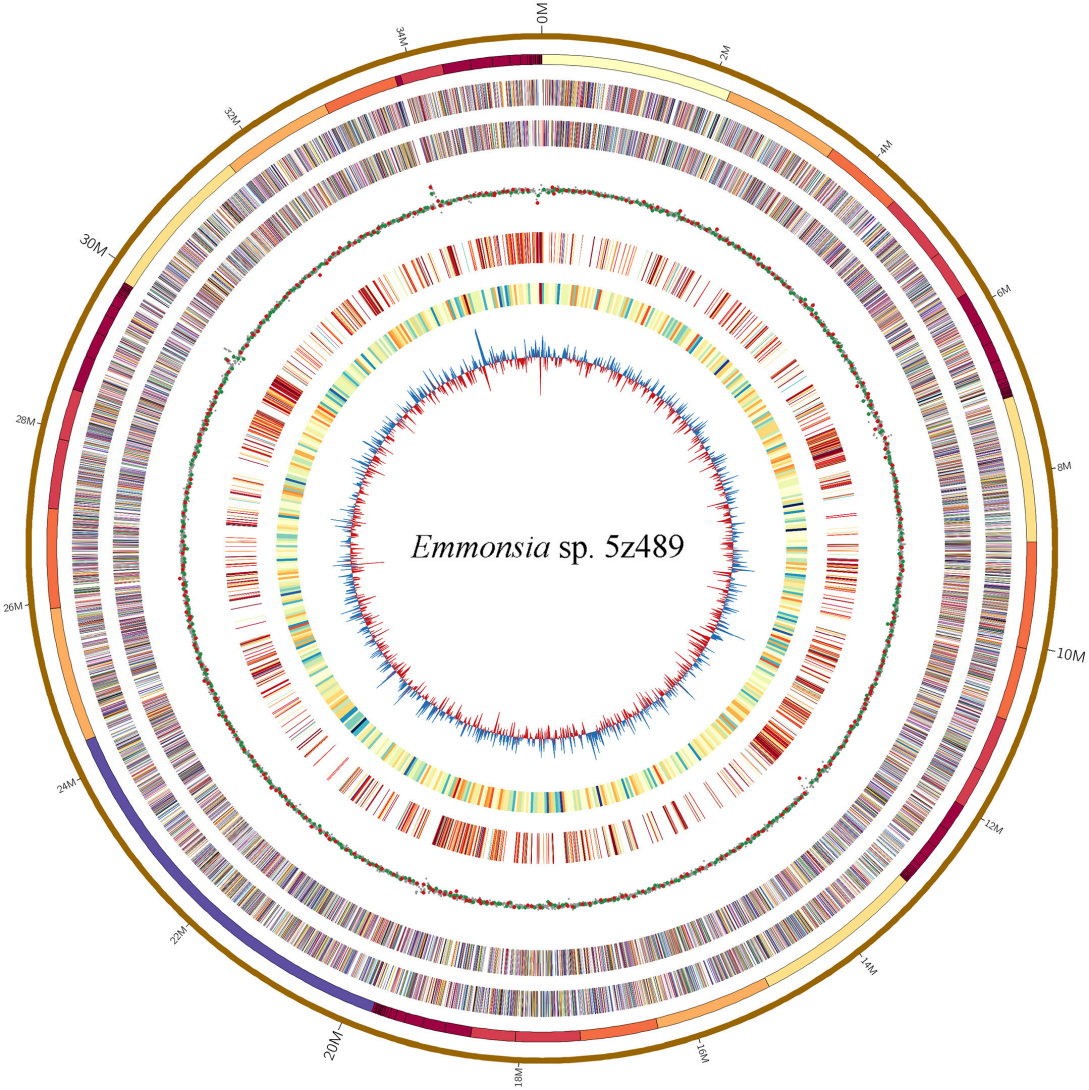
**Circular map of genomic features of *Emmonsia* sp. 5z489**. Circles are numbered from 1 (outermost) to 8 (innermost). Circles 1 and 2: a pseudo-genome of 35.5 Mbp and all the connected contigs. Circles 3 and 4: the locations of exons on the forward and reverse strands, respectively. Circle 5: global methylation patterns across the genome. Red dot, m6A; green dot, m4C; gray dot, type-uncertain modified base. Circle 6: repetitive elements across the genome. Circle 7: gene density delineated as the number of genes in 50-kbp non-overlapping windows. Circle 8: GC skew in 20-kbp non-overlapping windows.

The genome of *Emmonsia* sp. 5z489 was sequenced using a combination of the Pacific Biosciences RS II and Illumina MiSeq platforms. A total of 7.7 Gb and 5.9 Gb of raw data were generated with Pacbio sequencing and Illumina sequencing, respectively, with average coverage of 220x and 190x, respectively. The final assembly was ~35.5 Mbp, consisted of 108 contigs, and exhibited a contig N50 size of 1.3 Mbp. Read data from the Illumina platform was used to correct the assembled sequence generated with the PacBio platform in order to mitigate the relatively higher sequencing error rate of the latter (Ferrarini et al., 2013). In total, seven SNPs and 120 INDELs were corrected. To evaluate genome completeness, BLAST searches were performed using the core eukaryotic genes (CEGs) database. Of the 248 core eukaryotic genes in the database, 246 were identified, indicating > 99% genome coverage. The average gene density of 5z489 was 258 genes/Mbp, which is lower than the average gene density of other closely related species (Table 1). The average gene length was 1,631 bp and there was an average of 3.28 exons per gene. The overall GC content of the genome was 45.6%, which is consistent with other *Emmonsia* species (Table 1). The number of tRNA and rRNA genes (129 and 29, respectively) identified in the assembly were >2x that of other *Emmonsia* spp. genomes (Figure 1, Table 1). RepeatMasker was used to identify 4,459 repetitive elements in the *Emmonsia* sp. 5z489 genome, which covered 5,834,582 bp and represented ~16% of the total assembly. Repetitive elements consisted of 1,244 long interspersed nuclear elements (LINEs), 987 long terminal repeats (LTRs) and 288 DNA elements. However, 1,940 elements did not show homology to any existing repetitive sequences and were thus categorized as “unclassified.” Methylated adenine and cytosine bases were detected during PacBio sequencing. We found 3,006 N6-methyladenine (m6A) and 10,638 4-methylcytosine (m4C) base modifications distributed across the genome, with 417,110 type-uncertain modified bases during the analyses.

**Table 1:** **Genome features of *Emmonsia* sp. 5z489 and closely related species**.

**Feature**	***Emmonsia* sp. 5z489**	***E. crescens* UAMH 3008**	***E. parva* UAMH 139**	***Emmonsia* sp. CAC-2015a**
Size (Mb)	35.5	30.4	30.4	29.7
Coverage	190x (Illumina) & 220x (Pacbio)	163x	116x	29.4x
Sequencing technology	Illumina+Pacbio	Illumina	Illumina	IonTorrent
Contig #	108	2494	4901	4458
Contig N50 (kbp)	1300	40.1	19.4	13.5
(G + C)%	45.6	45.4	44.7	43.4
Protein-coding genes	9188	9558	8706	8860
Average gene length (bp)	1631.2	1365.5	1338.7	1339.7
Gene density (genes/Mbp)	258	310	280	298
Average exons per gene	3.28	2.88	2.89	2.87
Average exon length (bp)	495.8	473.9	462	466.1
Repeat (%)	16.4	5.4	9.9	10.4
tRNA	129	63	66	45
rRNA	29	8	10	3

In total, 9,188 protein-coding genes were predicted in the genome of 5z489, and 8,905 genes were functionally annotated based on their similarity to homologs in the NCBI non-redundant (nr) database (Supplementary Data 1). Additionally, GO terms were assigned to 4,079 protein-coding genes of 5z489 (44.4% of total genes). Almost 586 genes were assigned GO terms in the three primary categories: molecular function, biological process and cellular components. Additionally, 1,158, 338, and 112 genes exhibited GO terms that were unique to each category, respectively (Supplementary Figure 1, Supplementary Data 2). A total of 1,691 genes participating in 331 pathways were annotated using the KEGG database (Supplementary Data 3).

### Phylogenetic analysis

**Figure 2:**
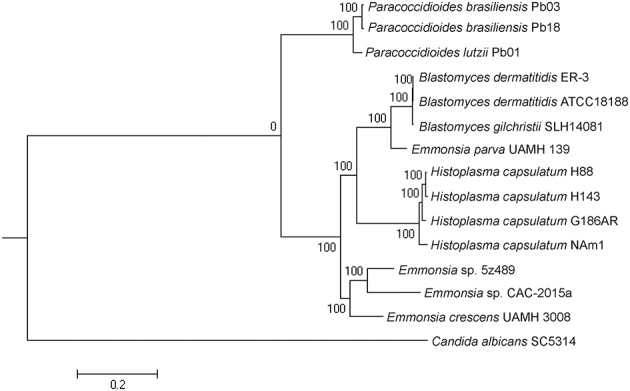
**Phylogeny of selected Ajellomycetaceae**. Maximum-likelihood phylogenetic tree of *Emmonsia* sp. 5z489 with other Ajellomycetaceae was constructed based on the sequences of 192 single-copy orthologs in RAxML. *Candida albicans* was used as an outgroup. Bootstrap values are indicated at nodes as a percentage of 1,000 bootstrap replicates.

Maximum-likelihood phylogenetic analysis in RAxML was conducted to assess the phylogenetic placement of *Emmonsia* sp. 5z489 among other members of the family Ajellomycetaceae with available whole-genome sequences. The analyses included genomes from three other *Emmonsia* strains, three *Paracoccidioides* strains, three *Blastomyces* strains, and four *Histoplasma* strains, with *Candida albicans* (family Saccharomycetaceae) used as an outgroup. In total, 192 single-copy orthologs were used for the phylogenetic analysis. As expected, phylogenetic analysis indicated that 5z489 clustered with *Emmonsia* species, separate from *E. parva*, which has previously been reported to group with the genus *Blastomyces* (Figure 2). Within the *Emmonsia* clade, strain UAMH 3008 was an out-group relative to 5z489 and CAC-2015a, both of which were only described recently. These results are consistent with recent phylogenetic analyses based on multiple sequences that showed that most novel *Emmonsia* strains form a single, derived clade in the family Ajellomycetaceae, with the exception of the first and the third novel *Emmonsia* isolates that affiliate with the *Blastomyces* (Schwartz et al., 2015b).

### Genomic comparisons with other *Emmonsia* species

**Figure 3:**
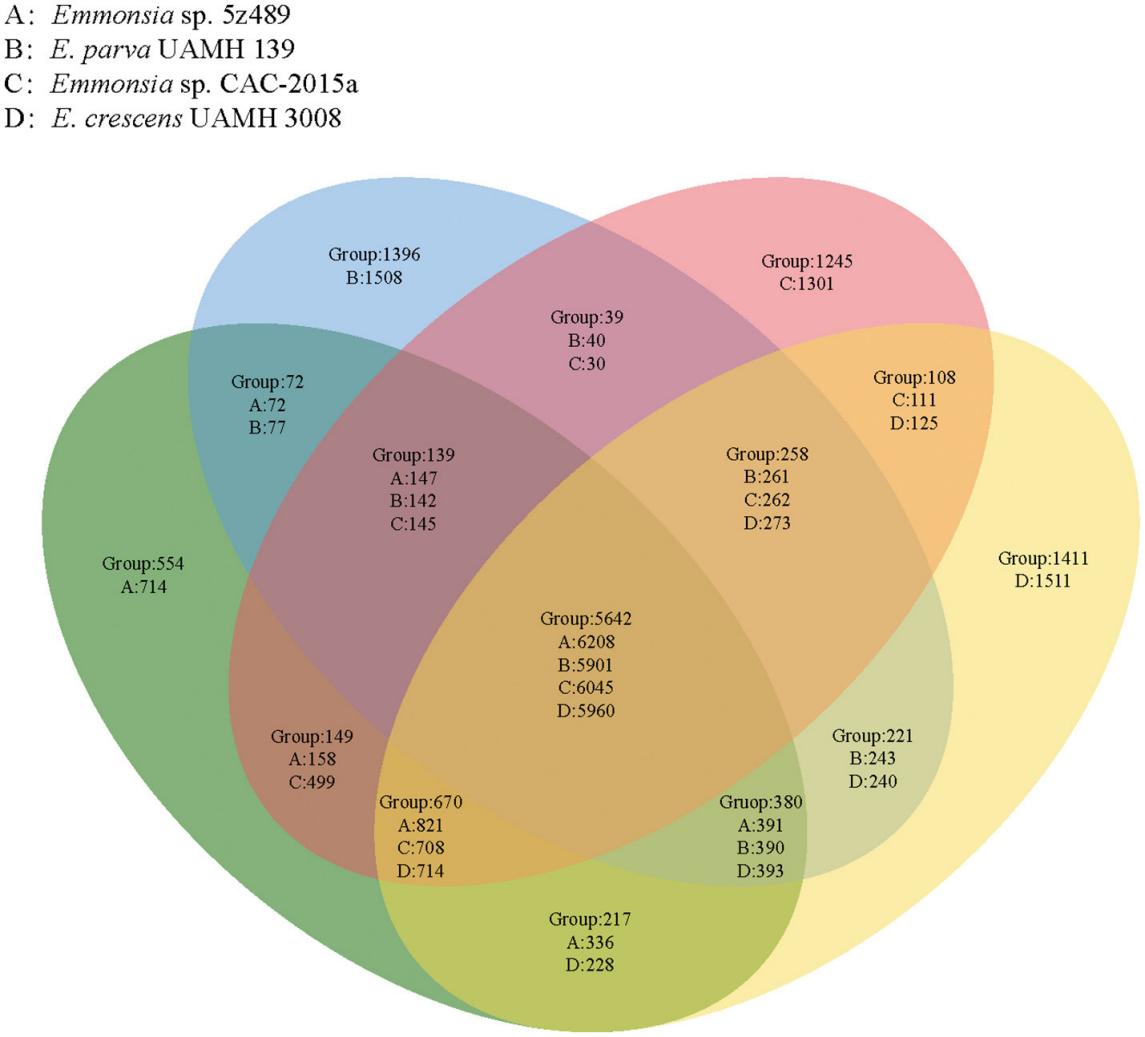
**Venn diagram showing unique and shared orthologous gene families between and among four *Emmonsia* species**. For each group, gene families are given for the entire group followed by the number of protein-coding genes unique to each group subset.

OrthoMCL analysis and analyses of shared proteins revealed that the four species of the genus *Emmonsia* analyzed here shared 5,642 gene families, which constitutes the vast majority of shared homologous genes among the four species. The second largest group of genes shared by these strains (*n* = 670 gene families) belonged to group A&C&D (*Emmonsia* sp. 5z489, *E. crescens* and *Emmonsia* sp. CAC-2015a), supporting the close relationship among these species to the exclusion of *E. parva*, which is consistent with the phylogenetic analysis. Group A (*Emmonsia* sp. 5z489) consisted of a number of unique paralog genes. Notably, this group had the least unique paralogous gene groups among all four species, including 554 gene families (714 protein-coding genes; Figure 3, Supplementary Data 4). CK1 protein kinases, serine/threonine protein kinases and zinc knuckle domain-containing proteins were more abundant in 5z489 than in the other species. CK1 protein kinases and serine/threonine protein kinases are known to regulate the majority of cellular pathways and especially those involved in signal transduction. To our knowledge, no members of this protein family have been documented to exhibit any virulent or pathogenic function. The zinc knuckle is a zinc-binding motif mostly associated with retroviral gag proteins and also includes members associated with eukaryotic gene regulation (Laity et al., 2001). Many zinc cluster (or binuclear) proteins, a class of zinc finger proteins, have been characterized to be involved in a wide range of processes in fungi, including stress response and pleiotropic drug resistance (MacPherson et al., 2006).

Pfam annotation was conducted to assign identity and functional annotation to 6,406 genes (70% of the total genes) of *Emmonsia* sp. 5z489 (Supplementary Data 5). The Pfam domains annotated for the 5z489 proteome were compared with the other three *Emmonsia* species (Supplementary Data 6). These comparisons revealed significantly higher enrichment of proteins in 5z489 containing F-box, Exo_endo_phos_2, gag_pre-integrs, rve, RVT_1, Helo_like_N, AAA, BCS1_N, RNase_H, RVT_2, Asp_protease_2, DUF3435, and DUF3723 families/domains. In addition, proteins possessing APH, Sugar_tr, TPR_10, Pectate_lyase_3, Pkinase, and Fungal_trans_2 families/domains were relatively more abundant in sp. 5z489.

No proteins with any Pfam families/domains were in obviously greater abundance in CAC-2015a relative to the other strains. Further, analysis showed that proteins with APH domains were in lower abundance in both 5z489 and CAC-2015a strains relative to those in *E. crescens*, while proteins with Pectate_lyase_3 domains were more enriched in these two strains. Proteins with APH domains belong to the phosphotransferase enzyme family, which consists of antibiotic resistance proteins that confer resistance to various aminoglycoside antibiotics (Sarwar and Akhtar, 1990; Trower and Clark, 1990). The low abundance of APH proteins in strains 5z489 and CAC-2015a likely indicates that they are sensitive to aminoglycosides and potentially informs the treatment of diseases caused by novel *Emmonsia* species. Pectate lyases have been isolated from several nematodes that parasitize plants (Doyle and Lambert, 2002; Huang et al., 2005; Kudla et al., 2007) and nematodes that are migratory and phytoparasitic (Kikuchi et al., 2006; Vanholme et al., 2007; Karim et al., 2009). The lyases are thought to soften and degrade the structure of plant cell walls during nematode migration and thus play key roles in the early stage of parasitism (Kudla et al., 2007; Davis et al., 2011; Peng et al., 2016). The abundance of Pectate_lyase_3 domains in novel *Emmonsia* isolate proteins indicates a potential role related to life style, for instance in nutrient uptake and/or invasion.

Eukaryotic Orthologous Groups (KOG) analysis revealed that *Emmonsia* sp. 5z489 contained greater gene enrichment within the “O” (Posttranslational modification, protein turnover, chaperones) and “R” (General function prediction only) categories, while the abundances within other categories were comparable to those of the other *Emmonsia* species, which indicates a general consilience of physiological and metabolic processes within this genus (Supplementary Figure 2, Supplementary Data 7).

### Gene families putatively involved in pathogenicity via the PHI database search

**Figure 4:**
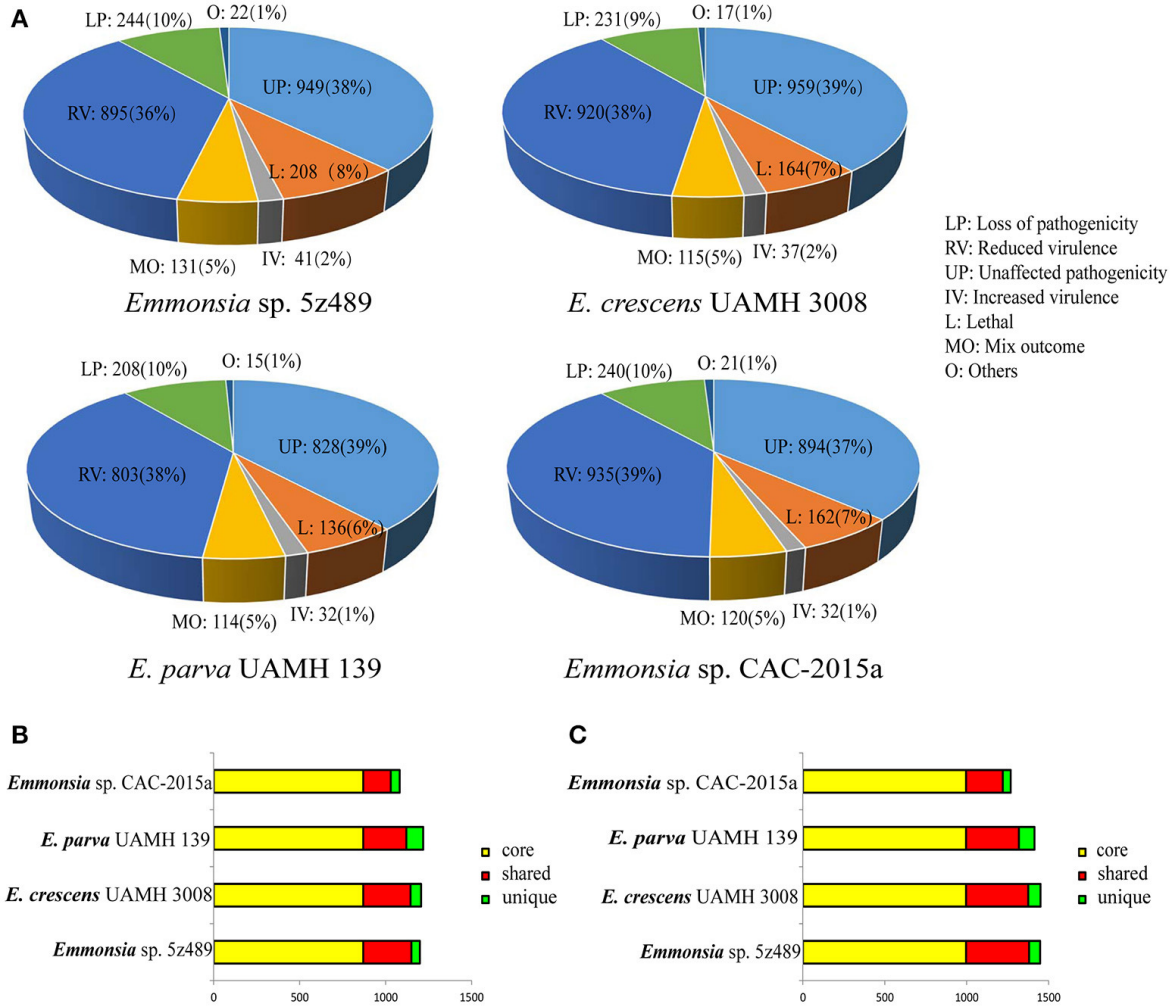
**Classification and orthology analysis of four *Emmonsia* species. (A)**. Classification of genes from each *Emmonsia* species based on the PHI database, with the numbers and proportions of genes indicated. **(B)**. Bar plot of orthology classes based on virulence and pathogenicity associated PHI genes. Core genes identified in all genomes are colored in yellow, shared genes present in more than one, but not all genomes, are colored in red, and genes unique to each genome are indicated in green. **(C)**. Bar plot of orthology classes based on the DFVF database. The color coding key is the same as in **(B)**.

To evaluate potential pathogenicity associated genes in *Emmonsia* sp. 5z489 and other *Emmonsia* species, genome-wide BLAST analysis against the Pathogen Host Interaction (PHI) database was performed. The PHI database is the first on-line resource devoted to the identification and presentation of information on pathogenicity genes and their host interactions (Winnenburg et al., 2006), and has been applied and cited in over 100 peer-reviewed publications (Urban et al., 2015). Protein-coding gene abundances identified as orthologs to PHI genes were similar among species and ranged from 2,136 in *E. parva* to 2,490 in 5z489. Each strain exhibited nearly equivalent ratios of genes related to virulence and pathogenicity (Figure 4A).

Virulence and pathogenicity associated PHI gene orthologs were then investigated among the four *Emmonsia* species. Numerous gene families (*n* = 870) were shared by all four strains, accounting for 58% of the total gene family clusters (Supplementary Data 8). Submaximal groups were shared by 5z489, UAMH 3008, and CAC-2015a (Figure 4B). This result is consistent with the phylogenetic position of *E. parva* as distinct from others in the genus *Emmonsia*. Moreover, this coincides with the putative pathogenic mechanism of *E. parva* appearing to be largely distinct from other *Emmonsia* species. A number of orthologs were identified that were shared among all four strains and included those with ABC transporter domains, aminotransferase domains, AMP-binding enzyme C-terminal (AMP-binding_C) domains, DnaJ domains, cytochromeP450 (CYP) families, Pkinase domains, Ras families, Major Facilitator Superfamily (MSF_1), as well as WD40 repeats. Proteins belonging to the cytochrome P450 or MSF_1 family are generally believed to play important roles in the biosynthesis and transportation of metabolites. Ras proteins are involved in cellular signal transduction as binary molecular switches. WD40 proteins are associated with numerous biological processes including signal transduction, cell division, cytoskeleton assembly, chemotaxis and RNA processing (Stirnimann et al., 2010). We deduce that several of these proteins may be involved in pathogenic processes that are common in all *Emmonsia*. In addition, 5z489 and CAC-2015a exhibited 49 and 98 unique gene groups, respectively (Supplementary Datas 9, 10), and some of these genes may contribute to differences in virulence and pathogenicity between strains. Furthermore, five orthologs of virulence and pathogenicity associated PHI genes were shared by 5z489 and CAC-2015a (Supplementary Data 11), and may contribute to the molecular basis of pathogenicity of novel strains which differs from classical *Emmonsia*.

### Gene families putatively involved in pathogenicity within the DFVF database

Since the repository of genes in the PHI database comprises numerous pathogenic microorganisms and their hosts, OrthoMCL analysis was performed using a fungal pathogen specific database, DFVF, to constrain the identification of fungal-specific virulence genes of the four *Emmonsia* species. Similar to the PHI analysis results, the majority of virulence gene groups were shared by all four strains. Additionally, the second greatest extent of gene group sharing was among *Emmonsia* spp. 5z489, UAMH 3008, and CAC-2015a, to the exclusion of *E. parva* (Figure 4C). In addition to orthologs identified in the PHI analysis with ABC transporter domains, AMP-binding_C domains, Pkinase domains, P450 families, MSF_1 families and WD40 repeats, proteins with sugar transporter families, SNF2 family N-terminal (SNF2_N) domains, fungal specific transcription factor (Fungal_trans) domains and DEAD/DEAH box helicase (DEAD) domains were found in much higher abundance in all *Emmonsia* species. Apart from sugar transporter family proteins, orthologs with other domains all exhibit various functions in RNA metabolism, suggesting that the regulation of these putative virulence factors at the transcriptional level may play a role in *Emmonsia* pathogenicity (Supplementary Data 12). 5z489 possessed 67 unique paralog gene groups (*n* = 133 genes), in which proteins with FAD binding domains were modestly abundant (Supplementary Data 13).

CAC-2015a possessed 96 unique gene groups (*n* = 96 genes), with proteins containing ABC_tran families, MFS_1 families and Pkinase domains being relatively abundant (Supplementary Data 14). Orthologs of DFVF genes that were shared by 5z489 and CAC-2015a were analyzed in order to assess the putative virulence factors common in novel *Emmonsia* and a total of 12 orthologs were found (Supplementary Data 15). Of these, proteins with RRM_1 domains comprised the majority. RNA Recognition Motif (RRM)-containing proteins are involved in rRNA processing, mRNA splicing, RNA editing, RNA stability, and translation (Ruwe et al., 2011; Zoschke et al., 2011; Wang et al., 2016).

### Putatively pathogenic gene families shared by the PHI and DFVF databases

Orthologs that are present in both the PHI and DFVF databases may be more likely to be related to pathogenicity than those only identified by either database. We therefore, integrated search results from both databases to identify shared orthologs. Thirteen shared orthologs were found in 5z489 (Supplementary Data 16) and 23 in CAC-2015a (Supplementary Data 17). The number of proteins with GMC_oxred_C domains is twice as high in 5z489 than any others. Glucose-methanol-choline oxidoreductase (GMC_oxred) family proteins are related to important developmental pathways (Takeuchi et al., 2005; Ahmad et al., 2006; Iida et al., 2007), chemical defenses (Rahfeld et al., 2014) and immunity (Sun et al., 2012) in higher eukaryotes. In fungi, GmcA, a member of GMC_oxred_C family, was first reported to be involved in the induction of asexual development in *Aspergillus nidulans* (Etxebeste et al., 2012). Proteins with ABC_trans domains exhibited lower abundance in CAC- 2015a relative to other *Emmonsia*. ATP-binding cassette transporters (ABC_tran) are found in all domains of life. In eukaryotes, most of these proteins are implicated in the transport of a variety of molecules across cellular membranes, and particularly in drug resistance via the export of specific toxic substances (Leprohon et al., 2011; Locher, 2016). These results suggest that several othologs that were identified in novel *Emmonsia*, and were shared by the PHI and DFVF databases, may play key roles in the life cycle and infectivity of these organisms.

### Secretory proteins associated with pathogenicity

Secretory proteins play crucial roles during the early infection of pathogenic fungi. We designed a comprehensive pipeline to predict secretory proteins of *Emmonsia* spp. 5z489 and CAC-2015a. The genome of 5z489 was predicted to encode 515 secreted proteins. The 515 putative secretory proteins were annotated using the PHI database and were further searched against the DFVF database. BLAST analyses indicated that 87 of the secretory proteins in 5z489 (accounting for 17% of the total secretome) were putatively involved in virulence and pathogenicity. Further, analysis suggested that two secretory proteins associated with virulence and pathogenicity were among the 714 total unique proteins in 5z489 (Table 2). Four hundred and ninety five secretory proteins were predicted in the CAC-2015a genome, and one of these is likely associated with virulence and pathogenicity and is also unique to strain CAC-2015a (Table 2).

**Table 2:** **Unique secretory proteins associated with virulence and pathogenicity in *Emmonsia* sp. 5z489 and CAC-2015a**.

**Gene ID**	**Pfam**	**Gene ontology**	**PHI accession**	**Phenotype**	**Description**	**DFVF uniprot ID**
5z489_g3837.t1	PF00026.21 Asp Family	GO:0006508 (proteolysis); GO:0004190 (aspartic-type endopeptidase activity)	PHI:3973	Reduced virulence	ASP	PEPA_ASPFU
5z489_g8410.t1	PF01476.18 LysM Domain; PF01476.18 LysM Domain; PF00187.17 Chitin_bind_1 Domain; PF00704.26 Glyco_hydro_18 Domain	GO:0005975 (carbohydrate metabolic process); GO:0004553 (hydrolase activity, hydrolyzing O-glycosyl compounds); GO:0008061 (chitin binding)	PHI:144	Reduced virulence	CHT42	O59928_HYPVI
sp_cac_rna1687	PF03198.12 Glyco_hydro_72 Domain		PHI:61	Reduced virulence	BGL2	GEL1_ASPFU

## Discussion

In this study, we obtained a 35.5 Mbp genome assembly of *Emmonsia* sp. 5z489. Interestingly, its assembly size is ~5 Mbp larger than other *Emmonsia* species. Further, 5.8 Mbp of repetitive elements were identified in 5z489, accounting for 16% of the genome. This abundance of repetitive elements is greater than both classical *Emmonsia* and strain CAC-2015a. Compared to other *Emmonsia* genomes that were generated solely by second-generation sequencing (SGS) methodologies, far fewer contigs of 5z489 were produced after read assembling, indicating a much higher level of genome quality, and completeness (Table 1). SGS technologies exhibit some shortcomings including shorter read lengths and limitations in the determination of repetitive regions, which may contribute to the differences seen in our assembly and those of other *Emmonsia* genomes (Muñoz et al., 2015). Single-Molecule Real-Time (SMRT) sequencing provides longer reads and more genomic information (e.g., DNA methylation patterns), but is hindered by a lower throughput and a higher error rate (Rhoads and Au, 2015). Since SGS and SMRT sequencing technologies both contain advantages and weaknesses, an innovative hybrid sequencing strategy that leverages the full advantages of both platforms has become more popular (Ritz et al., 2014; Yang et al., 2016).

DNA methylation plays a key role in numerous cellular processes including transcriptional repression, inactivation, embryonic development, genomic imprinting, the alteration of chromatin structure and transposon inactivation (Yong et al., 2016). Here, we detected 10,638 m4C bases and 3,006 m6A bases in the 5z489 genome. The identity of most modified bases (96.8%) is uncertain, but is likely to be 5-methylcytosine (m5C). The reasons for this supposition are as follows: (1) most eukaryotic DNA methyltransferases (MTases) are of the m5C class, and the most common epigenetic DNA modification is the methylation of cytosine leading to m5C (Clark et al., 2012; Laszlo et al., 2013), (2) in the absence of Tet1 oxidation treatment prior to SMRT sequencing, the kinetic signature of 5mC is subtler, making it difficult to be reliably detected (Flusberg et al., 2010; Clark et al., 2013). We further, examined the methylation distribution in the genome which indicated that 44% of the methylated bases were distributed across protein-coding genes, while 2.5% were located on repetitive elements. Interestingly, more than half of the modified bases were present in intergenic regions (Supplementary Table 1). It should be noted that this significantly differs from other fungi that have been investigated so far. In other fungi, methylation mostly occurs in repetitive loci that are responsible for silencing transponsable elements (TEs) and other repeats; with the exception of *Uncinocarpus reesii* that methylate both TEs and active genes (Lewis et al., 2009; Zemach et al., 2010). Genic DNA methylation in *Uncinocarpus reesii* is predicted to be directed by spliced mRNA (Zemach et al., 2010). However, these apparent differences may be due to differing sequence methodologies, because the DNA methylation patterns of most fungi were analyzed with bisulfite sequencing. Further, if the apparent differences are actually attributed to species-specific characteristics, it is yet unclear if this is related to the evolution of only *Emmonsia* species, as methylation data are not widely available for many other organisms. Regardless, these data suggest the need for further investigation.

It is pertinent to determine the phylogenetic position of novel, newly described *Emmonsia* in order to contextualize morphological and clinical observations. Here, the phylogenetic position was determined for the first time using whole-genome information for *Emmonsia* sp. 5z489, the seventh novel *Emmonsia*, and *Emmonsia* sp. CAC-2015a, the fifth novel *Emmonsia*. Within the family Ajellomycetaceae, the differentiation among *Emmonsia* appears to have occurred much earlier than in other genera, which contributes to relatively distant evolutionary relationships among species (Figure 2). This distinction may result from the underestimation of *Emmonsia* species diversity. Increased genome sequencing data will help elucidate if their diversity is truly underestimated or if the genus is indeed highly diverging relative to other genera of the Ajellomycetaceae. Our comparative analyses of virulence and pathogenicity associated genes also indicated that these two newly described *Emmonsia* species are more closely related to *E. crescens* than to *E. parva* (Figures 4B,C), which is consistent with the phylogenetic analyses. In addition, our results confirm the taxonomic classification of classical *Emmonsia* and accordingly, we suggest that *E. parva* should be formally classified into the genus *Blastomyces*.

The classical *Emmonsia*, within the family Ajellomycetaceae, along with *Blastomyces, Histoplasma*, and *Paracoccidioides*, all exhibit dimorphic shifts. *Emmonsia*, however, forms large, thick-walled adiaspores instead of yeast at high temperatures. A highly conserved histidine kinase, *DRK1*, is indispensable for dimorphism, virulence gene expression and pathogenicity in dimorphic fungi (Nemecek et al., 2006). A homolog of *DRK1* was also found in 5z489 (Gene ID: g8023.t1). In addition, the yeast phase-specific gene, *BYS1*, which is absent in both classical *Emmonsia* species, has been characterized as a phase transition marker to and from the yeast phase in *Blastomyces* (Burg and Smith, 1994). It is worth noting that a yeast phase specific protein was annotated in 5z489 (Gene ID: g3433.t1) which shared 35% amino acid identity to its homologous gene in *Histoplasma capsulatum*. Furthermore, we also identified orthologs of *DRK1* and *BYS1* in the genome of *Emmonsia* sp. CAC-2015a. These findings collectively suggest that regulation of phase transition in novel *Emmonsia* is more similar to other dimorphic fungi than to classical *Emmonsia* species. These results may partially explain why the clinical and histopathological characterizations of novel *Emmonsia* species invasion are more similar to blastomycosis or histoplasmosis than adiaspiromycosis.

Interestingly, compared to other *Emmonsia* spp., the 5z489 proteome displayed a much higher abundance of reverse transcriptase, retroviral integrase and RNase H families. We are presently not able to offer an explanation for this. However, it has been reported that reverse transcriptase activities are also associated with the replication of chromosome ends (telomerases) and some mobile genetic elements (retrotransposons), excluding a role in reverse transcription (Krieger et al., 2004). Two gene products unique to 5z489 were predicted to be secretory proteins associated with virulence and pathogenicity (Table 2). Aspartic-type endopeptidase activity was assigned to the first protein (Gene ID: g3837.t1) by GO term analysis, and Pfam annotation also suggested that it belongs to the eukaryotic aspartyl protease family. The second protein (Gene ID: g8410.t1) appeared to possess hydrolase activity as assigned by GO annotation and the Glyco_hydro_18 domain by Pfam annotation, suggesting that it may be an enzyme that functions in hydrolysing the glycosidic bonds between carbohydrates. In addition, one secretory protein (Gene ID: sp_cac_rna1687) was also suggested to be related to virulence and pathogenicity in CAC-2015a (Table 2). This protein is putatively involved in carbohydrate metabolic processes, and its function may be similar to that of pectate lyases. Although it is difficult to estimate the exact functions of these proteins without transcriptomic data or a biochemical investigation, it is reasonable to predict that they probably play a role in adaptation and pathogenicity to the host. They are thus potential targets for further investigations, including the therapeutic intervention of the newly described fungal pathogen.

## Conclusion

In this study we report the genome of *Emmonsia* sp. 5z489, a newly described *Emmonsia* pathogen, using a hybrid sequencing strategy. The assembly exhibited a much higher level of genome quality and completeness compared to other *Emmonsia* genomes. Additionally, genome screening provided data for global methylation patterns, with >50% of methylated bases located in intergenic regions, which represents a potentially unique genomic regulation feature compared to other fungi. This result represents a potential target for future exploration of the morphological and clinical differences that exist among novel pathogenic *Emmonsia*. Phylogenetic and comparative genomic analyses both support the classification of *Emmonsia* spp. 5z489 and CAC-2015a within the genus *Emmonsia*, revealed relatively distant evolutionary relationships among *Emmonsia*, and provided further evidence for the distinct evolutionary position of *E. parva*. Moreover, we provide baseline data on significant pathogenicity characteristics within the genus at the genome level, and putative virulence factors of novel *Emmonsia* strains, which may be potential targets for further research and therapeutic intervention of emmonsiosis. In conclusion, our study expands our understanding of the genomic features of *Emmonsia* species and provides a framework for understanding the evolutionary and pathogenic mechanisms underlying adaptations in these novel fungal pathogens.

## Author contributions

PW, JW, and HC conceived the project. YY and ZhL prepared the strain samples and performed sequence analyses. ZoL and XB conducted the bioinformatics analyses. YY, QY, and KL prepared the manuscript. YX and SW participated in discussions and provided suggestions. All authors read and approved the final manuscript.

## Funding

The study was supported in part by grants from the National Science and Technology Major Projects for “Major New Drugs Innovation and Development” of China (No. 2013ZX09304101) and the Award Funding for National Infrastructure of Microbial Resources of China (No. NIMR 2017-2).

### Conflict of interest statement

The authors declare that the research was conducted in the absence of any commercial or financial relationships that could be construed as a potential conflict of interest.
