# Supplementary material for: Genomics and Comparative Genomic Analyses Provide Insight into the Taxonomy and Pathogenic Potential of Novel Emmonsia Pathogens
Source: Front Cell Infect Microbiol. 2017 Mar 31;7:105. doi: 10.3389/fcimb.2017.00105 (PMC5374152; doi:10.3389/fcimb.2017.00105)
Supplement: Supplementary file 17 [file Table1.doc]

Supplementary Table 1. DNA methylation patterns across the genome of *Emmonsia* sp. 5z489.

| Type | Total | Distribution | |
| --- | --- | --- | --- |
|  |  | Genes | Repetitive sequnces |
| m4C | 10638 | 4514 (42%) | 883 (8%) |
| m6A | 3006 | 812 (27%) | 362 (12%) |
| m5C | 417110 | 185711 (44%) | 9505 (2%) |
