# Supplementary material for: Genomics and Comparative Genomic Analyses Provide Insight into the Taxonomy and Pathogenic Potential of Novel Emmonsia Pathogens
Source: Front Cell Infect Microbiol. 2017 Mar 31;7:105. doi: 10.3389/fcimb.2017.00105 (PMC5374152; doi:10.3389/fcimb.2017.00105)
Supplement: Supplementary file 18 [file Image1.PDF]

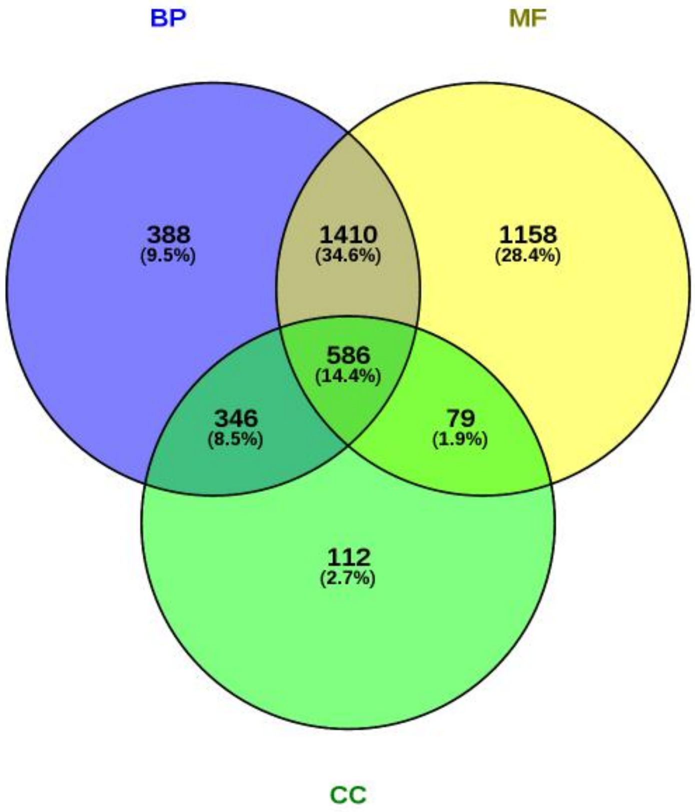

Supplementary Figure 1. The *Emmonsia* sp. 5z489 predicted genes assigned with Gene Ontology (GO) functional annotations. BLAST2GO software was used for GO classification based on similarity searches against the NCBI non-redundant database. BP, biological process; MF, molecular function; CC, cellular component.
