# Supplementary material for: Genomics and Comparative Genomic Analyses Provide Insight into the Taxonomy and Pathogenic Potential of Novel Emmonsia Pathogens
Source: Front Cell Infect Microbiol. 2017 Mar 31;7:105. doi: 10.3389/fcimb.2017.00105 (PMC5374152; doi:10.3389/fcimb.2017.00105)
Supplement: Supplementary file 19 [file Image2.PDF]

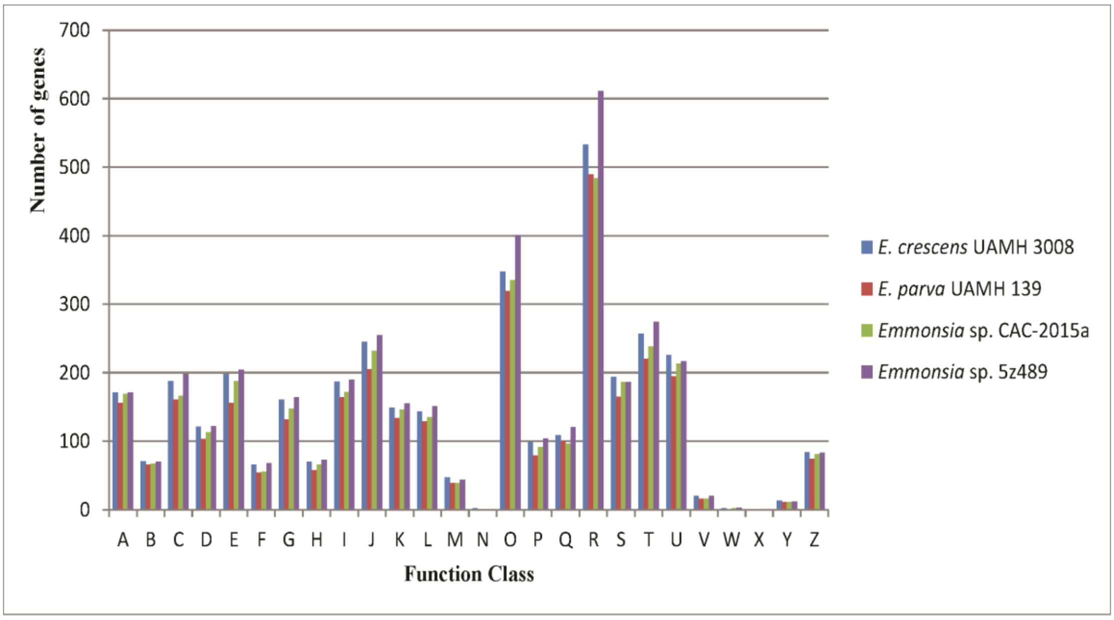

Supplementary Figure 2. The Eukaryotic Orthologous Groups (KOG) classification of *Emmonsia* species predicted genes. A, RNA processing and modification; B, Chromatin structure and dynamics; C, Energy production and conversion; D, Cell cycle control, cell division, chromosome partitioning; E, Amino acid transport and metabolism; F, Nucleotide transport and metabolism; G, Carbohydrate transport and metabolism; H, Coenzyme transport and metabolism; I, Lipid transport and metabolism; J, Translation, ribosomal structure and biogenesis; K, Transcription; L, Replication, recombination and repair; M, Cell wall/membrane/envelope biogenesis; N, Cell motility; O, Posttranslational modification, protein turnover, chaperones; P, Inorganic ion transport and metabolism; Q, Secondary metabolites biosynthesis, transport and catabolism; R, General function prediction only; S, Function unknown; T, Signal transduction mechanisms; U, Intracellular trafficking, secretion, and vesicular transport; V, Defense mechanisms; W,

Extracellular structures; X, Mobilome: prophages, transposons; Y, Nuclear structure; Z, Cytoskeleton.
